# 38 Combining healthcare and fitness through physical co-location: is that alone enough to get people physically active?

**DOI:** 10.1093/eurpub/ckae114.154

**Published:** 2024-09-26

**Authors:** Natalie Grinvalds, Robert J Copeland, Katie Shearn, Helen Humphreys

**Affiliations:** Sheffield Hallam University Advanced Wellbeing Research Centre, Sheffield, United Kingdom; National Centre for Sport and Exercise Medicine, Sheffield, United Kingdom; Sheffield Hallam University Advanced Wellbeing Research Centre, Sheffield, United Kingdom; National Centre for Sport and Exercise Medicine, Sheffield, United Kingdom; National Centre for Sport and Exercise Medicine, Sheffield, United Kingdom; National Centre for Sport and Exercise Medicine, Sheffield, United Kingdom

## Abstract

The primary purpose of this research was to explain how co-location of healthcare (community or secondary healthcare clinics) with fitness centres (gyms, leisure centres) works (or does not work) to promote and facilitate access to physical activity opportunities and physical activity behaviour. As part of 2012 London Olympic Legacy and National Centre for Sport & Exercise Medicine, National Health Service (NHS) clinics were co-located with leisure centres. Aims of this initiative were to promote PA as prevention and treatment through routine healthcare, increase awareness, normalise PA, and bring healthcare out of hospitals and into the community. Despite numerous calls for co-location of healthcare in alternative settings, little empirical evidence exists to show how fitness centres work as an appropriate environment.

This work is innovative topically as it is the first study (at time of publication) to research co-location of healthcare and fitness in UK and methodologically novel for the use of realist review and evaluation to develop a set of theories to explain co-location.

As part of a two-phase realist evaluation, phase 2 of this research resulted in theories and evidence to show how, why, for whom and under what circumstances co-location works (or not). Theories developed in Phase 1 were ‘tested’ through interviews with ten healthcare professionals and ten patients in four clinical services (including diabetes, pain management, musculoskeletal physiotherapy, podiatry) in co-located sites.

Five refined programme theories were developed suggesting that co-location works for people living with LTCs who are motivated to be active but need support. Co-location of health and fitness works for healthcare professionals (HCPs) who are active, knowledgeable, make time to discuss PA with patients. Co-location creates a salutogenic environment which enables patients and HCPs to become active. Enabling contexts include aligned business models, shared clinical and PA scheduling and teamwork between HCPs and exercise professionals. Logistical challenges and individual motivations are barriers to co-location working to promote PA.

Co-location of healthcare and fitness, with optimal implementation, can result in promotion of PA through healthcare, enabling people become physically active and reduce the burden of LTCs on society.

Ukactive and a SHU Vice Chancellor’s scholarship

